# Natural Products in Precision Oncology: Plant-Based Small Molecule Inhibitors of Protein Kinases for Cancer Chemoprevention

**DOI:** 10.3390/nu15051192

**Published:** 2023-02-27

**Authors:** Henry J. Thompson, Tymofiy Lutsiv

**Affiliations:** 1 Cancer Prevention Laboratory, Colorado State University, Fort Collins, CO 80523, USA; 2 Graduate Program in Cell & Molecular Biology, Colorado State University, Fort Collins, CO 80523, USA

**Keywords:** cancer kinome, chemoprevention, natural products, small molecule inhibitors, precision oncology

## Abstract

Striking progress is being made in cancer treatment by using small molecule inhibitors of specific protein kinases that are products of genes recognized as drivers for a specific type of cancer. However, the cost of newly developed drugs is high, and these pharmaceuticals are neither affordable nor accessible in most parts of the world. Accordingly, this narrative review aims to probe how these recent successes in cancer treatment can be reverse-engineered into affordable and accessible approaches for the global community. This challenge is addressed through the lens of cancer chemoprevention, defined as using pharmacological agents of natural or synthetic origin to impede, arrest, or reverse carcinogenesis at any stage in the disease process. In this regard, prevention refers to reducing cancer-related deaths. Recognizing the clinical successes and limitations of protein kinase inhibitor treatment strategies, the disciplines of pharmacognosy and chemotaxonomy are juxtaposed with current efforts to exploit the cancer kinome to describe a conceptual framework for developing a natural product-based approach for precision oncology.

## 1. Introduction

*Cancer* is a leading cause of death worldwide, with 10 million deaths reported in the year 2020, and the most significant obstacle to increasing life expectancy in every country in the world in the 21st century [1,2]. Cancer is a generic term that applies to a large and heterogeneous array of diseases (over a hundred) originating from the transformation of a normal cell through multiple steps into one with an abnormal and deregulated capacity to divide (clonal growth advantage) and invade adjacent ones and eventually remote tissues, thereby affecting almost every organ and part of the body [1]. By recognizing the physical, psychosocial, and financial burden that cancer generates globally for individuals, communities, and health systems, the essentiality of cancer prevention is re-emphasized [3,4]. Today, up to a half of all cancers can be prevented not only through evasion of known cancer risk factors but also via active and multifaceted surveillance and intervention efforts [1,5]. These efforts fall under the umbrella of *precision oncology* which is defined as cancer diagnosis, prognosis, prevention and/or treatment tailored specifically to the individual patient based on the genetic and/or molecular profile of the patient [6]. Furthermore, emerging transformative successes in cancer treatment can be leveraged for additional evidence-based cancer prevention strategies, as we have recently posited [5].

Historically, the development of cytotoxic chemotherapeutic regimes was the primary focus of clinical cancer research. In 1976, however, a member of the National Cancer Institute, Bethesda, MD, USA, Michael B. Sporn coined the term *chemoprevention* [7,8]. Conceptionally, the perception of cancer not as a clinical endpoint, but rather as a disease process, i.e., carcinogenesis and the realization that efforts must be made to target every step of this process are the key distinctions between the chemopreventive versus chemotherapeutic strategies. Therefore, chemoprevention embodies the utilization of chemical substances of natural or synthetic origin to impede, arrest, or reverse carcinogenesis at any stage in the disease process enhancing escape from cancer-related comorbidities and reducing the number of cancer-associated deaths. Within the following five decades since the term chemoprevention was coined, other names have been advanced to emphasize various dimensions of prevention strategies that fall under the umbrella of chemoprevention, e.g., chemoprotection, chemoprophylaxis, and chemo-interference; however, their conceptual basis remains the same.

The first gene with carcinogenic potential that was identified to drive the transformation of a cell encoded SRC protein —a member of the protein kinase superfamily of enzymes that regulate numerous cellular functions involved in the development of cancer [9]. Subsequently, protein kinases have become primary targets of drug discovery in the 21st century, and their inhibitors constitute about a third of all efforts to develop pharmacological agents both in the United States and across the globe [10]. However, the amount of resources and the cost necessary to develop and distribute these novel drugs is high. Considering that the income level of the countries has been a key factor contributing to substantial variation in the availability of cancer treatment, whereby more than 90% of high-income and barely 15% of low-income countries can successfully access comprehensive treatment options [11], most of the target drugs are not accessible in the majority of the world. Therefore, this narrative review aims to probe how recent progress in cancer treatment targeting protein kinases can be reverse-engineered into affordable and accessible approaches for the global community under the umbrella of natural product-based precision oncology.

Identifying the sources of natural products that can be developed into drugs is the traditional goal of pharmacognosy [12], and plants have been historically utilized as sources of natural compounds with therapeutic properties of diverse chemical characteristics [13,14]. Unsurprisingly, the latter have been used in one of the relatively modern approaches to classify plants—chemotaxonomy [15]. Thus, the rationale underlying a focus on protein kinase inhibitors in this review is four-fold: (1) specific kinases have been identified as drivers for each major type of cancer, and these kinases become deregulated at multiple steps in the carcinogenic process; (2) successes in targeting the cancer kinome, i.e., the network of protein kinases in the cell [9,16], provide a framework for robust investigation of naturally-sourced small molecule inhibitors for specific kinases as well as an understanding of the limitations of their inhibition; (3) a focus on the disciplines of pharmacognosy and chemotaxonomy provides a botanical paradigm for aligning plant sources of kinase inhibitors with the protein kinases that need to be targeted to intervene in specific cancer types; and (4) in many countries around the world, the medicinal use of plants and natural products derived from them is culturally accepted.

## 2. Targeting the Kinome with Natural Products Originating in Plants

Of the many druggable targets that could be selected, protein kinases are considered of particular value owing to their role in intracellular signaling pathways, dysregulation of which is involved in the pathogenesis of numerous chronic diseases, including but not limited to nervous, cardiovascular, inflammatory, autoimmune, metabolic disorders, type-2 diabetes, and cancer [10,17]. Interest in the role of protein kinases in health and disease dates back to the 1950′s when the first enzymatic phosphorylation reaction was demonstrated for casein by phosphorylase kinase [16,18]. Protein kinases catalyze a reversible reaction of phosphorylation of target proteins (substrates), whereby negatively charged terminal γ-phosphoryl group (PO_3_^2−^) is transferred from adenosine triphosphate (ATP) and covalently attached to the free hydroxyl (-OH) group on an amino acid (phosphorylation site) of pre-existing target protein in the context of its post-translational modification, altering its conformation and, therefore, function. The nature of the amino acid that is to be phosphorylated determines the classification of protein kinases: serine/threonine-specific protein kinases consist of 385 members; tyrosine-specific protein kinases consist of 58 transmembrane receptors and 32 intracellular non-receptor proteins; tyrosine kinase-like enzymes consist of 43 members; a small group of dual specificity protein-kinases phosphorylating both tyrosine and threonine residues; and others [10,19]. Alternatively, eukaryotic protein kinases are clustered into ten groups based on their sequence similarity in the kinase domain, evolutionary conservation, and functionality [19]. At this time, the human kinome is estimated to consist of 538 genes [20]. Altogether, 2.5% of human protein-coding genes reportedly comprise protein kinases, half localized within cancer or other disease loci [10].

**Figure 1:**
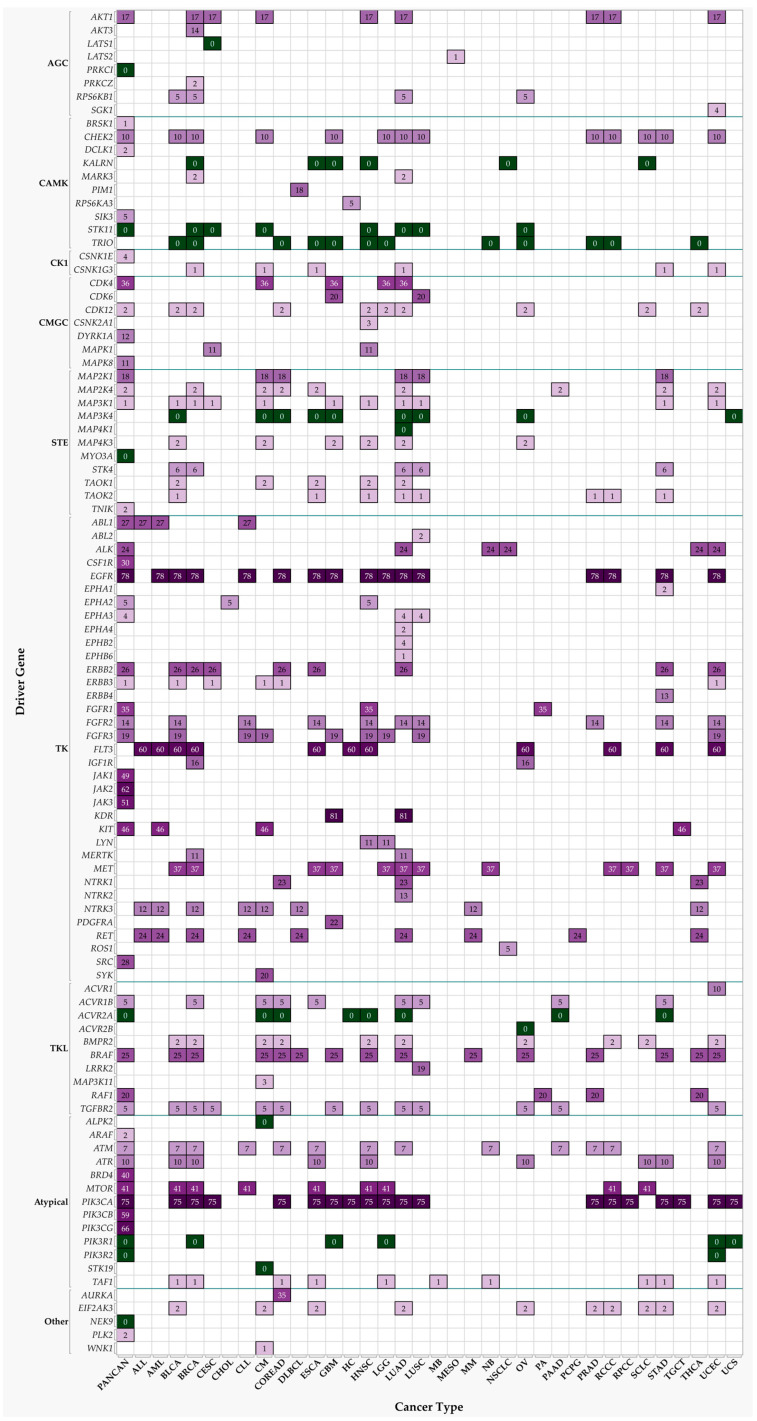
Protein kinase drivers of various cancer types as targets for pharmacological inhibitor development. Heatmap depicts established associations of protein kinases as drivers of certain types of cancer, including those that are additionally targets of active inhibitor drug development [9,27,30]. Numbers indicate amounts of available pharmacological inhibitors or negative allosteric modulators, also depicted in shades of purple, while green indicates a lack thereof. ALL: Acute Lymphocytic Leukemia; AML: Acute Myeloid Leukemia; BLCA: Bladder Carcinoma; BRCA: Breast Carcinoma; CESC: Cervical squamous cell carcinoma and endocervical adenocarcinoma; CHOL: Cholangiocarcinoma; CLL: Chronic Lymphocytic Leukemia; CM: Cutaneous Melanoma; COREAD: Colorectal Adenocarcinoma; DLBC: Lymphoid Neoplasm Diffuse Large B-cell Lymphoma; ESCA: Esophageal Carcinoma; GBM: Glioblastoma; HC: Hepatocellular Carcinoma; HNSC: Head and Neck squamous cell carcinoma; LGG: Lower Grade Glioma; LUAD: Lung Adenocarcinoma; LUSC: Lung Squamous Cell Carcinoma; MB: Medulloblastoma; MESO: Mesothelioma; MM: Multiple Myeloma; NB: Neuroblastoma; NSCLC: Non-Small-Cell Lung Cancer; OV: Serous Ovarian Adenocarcinoma; PA: Pylocytic Astrocytoma; PAAD: Pancreatic Ductal Adenocarcinoma; PANCAN: PanCancer study; PCPG: Pheochromocytoma and Paraganglioma; PRAD: Prostate Adenocarcinoma; RPCC: Renal Papillary Cell Carcinoma; RCCC: Renal Clear Cell Carcinoma; SCLC: Small-Cell Lung Cancer; STAD: Stomach Adenocarcinoma; TGCT: Testicular Germ Cell Tumors; THCA: Thyroid Carcinoma; UCEC: Uterine Corpus Endometrioid Carcinoma; UCS: Uterine Carcinosarcoma; ABL1: ABL proto-oncogene 1, non-receptor tyrosine kinase; ABL2: ABL proto-oncogene 2, non-receptor tyrosine kinase; ACVR1: activin A receptor type 1; ACVR1B: activin A receptor type 1B; ACVR2A: activin A receptor type 2A; ACVR2B: activin A receptor type 2B; AKT1: AKT serine/threonine kinase 1; AKT3: AKT serine/threonine kinase 3; ALK: ALK receptor tyrosine kinase; ALPK2: alpha kinase 2; ARAF: A-Raf proto-oncogene, serine/threonine kinase; ATM: ATM serine/threonine kinase; ATR: ATR serine/threonine kinase; AURKA: aurora kinase A; BMPR2: bone morphogenetic protein receptor type 2; BRAF: B-Raf proto-oncogene, serine/threonine kinase; BRD4: bromodomain containing 4; BRSK1: BR serine/threonine kinase 1; CDK12: cyclin dependent kinase 12; CDK4: cyclin dependent kinase 4; CDK6: cyclin dependent kinase 6; CHEK2: checkpoint kinase 2; CSF1R: colony stimulating factor 1 receptor; CSNK1E: casein kinase 1 epsilon; CSNK1G3: casein kinase 1 gamma 3; CSNK2A1: casein kinase 2 alpha 1; DCLK1: doublecortin like kinase 1; DYRK1A: dual specificity tyrosine phosphorylation regulated kinase 1A; EGFR: epidermal growth factor receptor; EIF2AK3: eukaryotic translation initiation factor 2 alpha kinase 3; EPHA1: EPH receptor A1; EPHA2: EPH receptor A2; EPHA3: EPH receptor A3; EPHA4: EPH receptor A4; EPHA6: EPH receptor A6; EPHB2: EPH receptor B2; EPHB6: EPH receptor B6; ERBB2: erb-b2 receptor tyrosine kinase 2; ERBB3: erb-b2 receptor tyrosine kinase 3; ERBB4: erb-b2 receptor tyrosine kinase 4; FGFR1: fibroblast growth factor receptor 1; FGFR2: fibroblast growth factor receptor 2; FGFR3: fibroblast growth factor receptor 3; FLT3: fms related receptor tyrosine kinase 3; IGF1R: insulin like growth factor 1 receptor; JAK1: Janus kinase 1; JAK2: Janus kinase 2; JAK3: Janus kinase 3; KALRN: kalirin RhoGEF kinase; KDR: kinase insert domain receptor; KIT: KIT proto-oncogene, receptor tyrosine kinase; LATS1: large tumor suppressor kinase 1; LATS2: large tumor suppressor kinase 2; LRRK2: leucine rich repeat kinase 2; LYN: LYN proto-oncogene, Src family tyrosine kinase; MAP2K1: mitogen-activated protein kinase kinase 1; MAP2K4: mitogen-activated protein kinase kinase 4; MAP3K1: mitogen-activated protein kinase kinase kinase 1; MAP3K11: mitogen-activated protein kinase kinase kinase 11; MAP3K4: mitogen-activated protein kinase kinase kinase 4; MAP4K1: mitogen-activated protein kinase kinase kinase kinase 1; MAP4K3: mitogen-activated protein kinase kinase kinase kinase 3; MAPK1: mitogen-activated protein kinase 1; MAPK8: mitogen-activated protein kinase 8; MARK3: microtubule affinity regulating kinase 3; MERTK: MER proto-oncogene, tyrosine kinase; MET: MET proto-oncogene, receptor tyrosine kinase; MTOR: mechanistic target of rapamycin kinase; MYO3A: myosin IIIA; NEK9: NIMA related kinase 9; NTRK1: neurotrophic receptor tyrosine kinase 1; NTRK2: neurotrophic receptor tyrosine kinase 2; NTRK3: neurotrophic receptor tyrosine kinase 3; PDGFRA: platelet derived growth factor receptor alpha; PIK3CA: phosphatidylinositol-4,5-bisphosphate 3-kinase catalytic subunit alpha; PIK3CB: phosphatidylinositol-4,5-bisphosphate 3-kinase catalytic subunit beta; PIK3CG: phosphatidylinositol-4,5-bisphosphate 3-kinase catalytic subunit gamma; PIK3R1: phosphoinositide-3-kinase regulatory subunit 1; PIK3R2: phosphoinositide-3-kinase regulatory subunit 2; PIM1: Pim-1 proto-oncogene, serine/threonine kinase; PLK2: polo like kinase 2; PRKCI: protein kinase C iota; PRKCZ: protein kinase C zeta; RAF1: Raf-1 proto-oncogene, serine/threonine kinase; RET: ret proto-oncogene; ROS1: ROS proto-oncogene 1, receptor tyrosine kinase; RPS6KA3: ribosomal protein S6 kinase A3; RPS6KB1: ribosomal protein S6 kinase B1; SGK1: serum/glucocorticoid regulated kinase 1; SIK3: SIK family kinase 3; SRC: SRC proto-oncogene, non-receptor tyrosine kinase; STK11: serine/threonine kinase 11; STK19: serine/threonine kinase 19; STK4: serine/threonine kinase 4; SYK: spleen associated tyrosine kinase; TAF1: TATA-box binding protein associated factor 1; TAOK1: TAO kinase 1; TAOK2: TAO kinase 2; TGFBR2: transforming growth factor beta receptor 2; TNIK: TRAF2 and NCK interacting kinase; TRIO: trio Rho guanine nucleotide exchange factor; and WNK1: WNK lysine deficient protein kinase 1.

The resulting modified proteins alter their enzymatic activity (especially, if the substrate is another protein kinase participating amplifying signal transduction towards their own downstream targets), change their intracellular localization, their turnover, or acquire/lose the ability to interact with other proteins. Protein kinases regulate various cellular physiological events, among which there are cell cycle control and progression, response to extracellular stimuli, cell survival, apoptosis, differentiation, metabolic control, migration, and DNA damage response [10,17,21]. These are among the key cellular functions, the disruption of which contributes to the development of cancer [22,23,24]. Genes whose mutational alteration results in dysfunction of their protein products grant the selective net advantage to a cell to collectively acquire diverse hallmark capabilities of cancer [22,23,24], i.e., drive the progressive transformation of a normal cell into a malignant one; they are called *drivers* [25,26]. There are 102 protein kinases identified as drivers of various types of cancer [9,27]. Mutations in these genes were found to be associated with the key oncogenic events in The Cancer Genome Atlas (TCGA) projects of various cancer types as well as in the Pan-Cancer studies. Many of these protein kinases have been targets of pharmacological inhibition and respective drug development in the realms of cancer treatment that were approved by the United States Food and Drug Administration (USFDA) [10,28,29]. All this information is also summarized in Figure 1 and Supplementary Tables S1 and S2, quantifying the number of drugs developed for particular protein kinases. The information about available pharmacological inhibitors was obtained from the International Union of Basic and Clinical Pharmacology (IUPHAR)/British Pharmacological Society (BPS) Guide to PHARMACOLOGY [30] and provided in the Supplementary Table S3. Among these, protein kinase inhibitors have been most actively developed for kinase insert domain receptor (KDR, also known as vascular endothelial growth factor receptor 2, VEGFR-2), epidermal growth factor receptor (EGFR), phosphatidylinositol-4,5-bisphosphate 3-kinase catalytic subunits (PIK3CA/B/G), Janus kinases (JAK1/2/3), and Fms related receptor tyrosine kinase 3 (FLT3).

**Figure 2:**
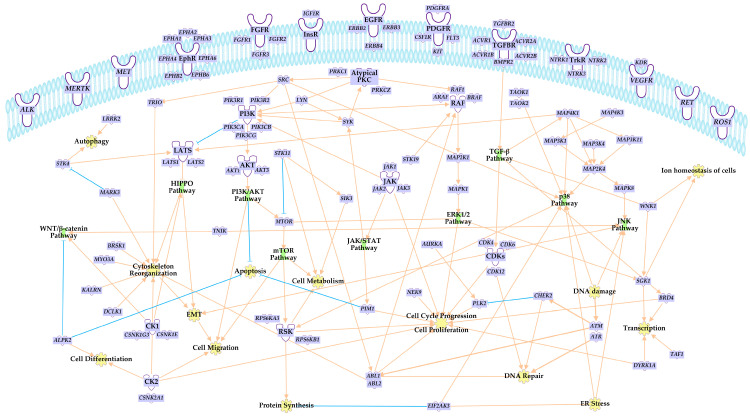
Drivers of cancer development within cellular kinome. The scheme represents established genes coding for protein kinases and their intracellular signaling roles, disruption of which is involved in chronic disease. The figure was compiled using information provided in the literature [9,17,22,23,24,27] as well as QIAGEN Ingenuity Pathway Analysis Knowledge Base. On the plasma membrane (top), there are tyrosine kinase and tyrosine kinase-like receptors, the activation of which initiates various signaling cascades depicted inside the cell. For simplification, only the driver genes are included in the figure; the relationship arrow lines indicate activation/causation (in light orange) or inhibition/suppression (in light blue); only the most prominent intracellular signaling pathways (in green) and cellular events associated with respective kinases (in yellow) are depicted. ABL1: ABL proto-oncogene 1, non-receptor tyrosine kinase; ABL2: ABL proto-oncogene 2, non-receptor tyrosine kinase; ACVR1: activin A receptor type 1; ACVR1B: activin A receptor type 1B; ACVR2A: activin A receptor type 2A; ACVR2B: activin A receptor type 2B; AKT1: AKT serine/threonine kinase 1; AKT3: AKT serine/threonine kinase 3; ALK: ALK receptor tyrosine kinase; ALPK2: alpha kinase 2; ARAF: A-Raf proto-oncogene, serine/threonine kinase; ATM: ATM serine/threonine kinase; ATR: ATR serine/threonine kinase; AURKA: aurora kinase A; BMPR2: bone morphogenetic protein receptor type 2; BRAF: B-Raf proto-oncogene, serine/threonine kinase; BRD4: bromodomain containing 4; BRSK1: BR serine/threonine kinase 1; CDK12: cyclin dependent kinase 12; CDK4: cyclin dependent kinase 4; CDK6: cyclin dependent kinase 6; CHEK2: checkpoint kinase 2; CSF1R: colony stimulating factor 1 receptor; CSNK1E: casein kinase 1 epsilon; CSNK1G3: casein kinase 1 gamma 3; CSNK2A1: casein kinase 2 alpha 1; DCLK1: doublecortin like kinase 1; DYRK1A: dual specificity tyrosine phosphorylation regulated kinase 1A; EGFR: epidermal growth factor receptor; EIF2AK3: eukaryotic translation initiation factor 2 alpha kinase 3; EPHA1: EPH receptor A1; EPHA2: EPH receptor A2; EPHA3: EPH receptor A3; EPHA4: EPH receptor A4; EPHA6: EPH receptor A6; EPHB2: EPH receptor B2; EPHB6: EPH receptor B6; ERBB2: erb-b2 receptor tyrosine kinase 2; ERBB3: erb-b2 receptor tyrosine kinase 3; ERBB4: erb-b2 receptor tyrosine kinase 4; FGFR1: fibroblast growth factor receptor 1; FGFR2: fibroblast growth factor receptor 2; FGFR3: fibroblast growth factor receptor 3; FLT3: fms related receptor tyrosine kinase 3; IGF1R: insulin like growth factor 1 receptor; JAK1: Janus kinase 1; JAK2: Janus kinase 2; JAK3: Janus kinase 3; KALRN: kalirin RhoGEF kinase; KDR: kinase insert domain receptor; KIT: KIT proto-oncogene, receptor tyrosine kinase; LATS1: large tumor suppressor kinase 1; LATS2: large tumor suppressor kinase 2; LRRK2: leucine rich repeat kinase 2; LYN: LYN proto-oncogene, Src family tyrosine kinase; MAP2K1: mitogen-activated protein kinase kinase 1; MAP2K4: mitogen-activated protein kinase kinase 4; MAP3K1: mitogen-activated protein kinase kinase kinase 1; MAP3K11: mitogen-activated protein kinase kinase kinase 11; MAP3K4: mitogen-activated protein kinase kinase kinase 4; MAP4K1: mitogen-activated protein kinase kinase kinase kinase 1; MAP4K3: mitogen-activated protein kinase kinase kinase kinase 3; MAPK1: mitogen-activated protein kinase 1; MAPK8: mitogen-activated protein kinase 8; MARK3: microtubule affinity regulating kinase 3; MERTK: MER proto-oncogene, tyrosine kinase; MET: MET proto-oncogene, receptor tyrosine kinase; MTOR: mechanistic target of rapamycin kinase; MYO3A: myosin IIIA; NEK9: NIMA related kinase 9; NTRK1: neurotrophic receptor tyrosine kinase 1; NTRK2: neurotrophic receptor tyrosine kinase 2; NTRK3: neurotrophic receptor tyrosine kinase 3; PDGFRA: platelet derived growth factor receptor alpha; PIK3CA: phosphatidylinositol-4,5-bisphosphate 3-kinase catalytic subunit alpha; PIK3CB: phosphatidylinositol-4,5-bisphosphate 3-kinase catalytic subunit beta; PIK3CG: phosphatidylinositol-4,5-bisphosphate 3-kinase catalytic subunit gamma; PIK3R1: phosphoinositide-3-kinase regulatory subunit 1; PIK3R2: phosphoinositide-3-kinase regulatory subunit 2; PIM1: Pim-1 proto-oncogene, serine/threonine kinase; PLK2: polo like kinase 2; PRKCI: protein kinase C iota; PRKCZ: protein kinase C zeta; RAF1: Raf-1 proto-oncogene, serine/threonine kinase; RET: ret proto-oncogene; ROS1: ROS proto-oncogene 1, receptor tyrosine kinase; RPS6KA3: ribosomal protein S6 kinase A3; RPS6KB1: ribosomal protein S6 kinase B1; SGK1: serum/glucocorticoid regulated kinase 1; SIK3: SIK family kinase 3; SRC: SRC proto-oncogene, non-receptor tyrosine kinase; STK11: serine/threonine kinase 11; STK19: serine/threonine kinase 19; STK4: serine/threonine kinase 4; SYK: spleen associated tyrosine kinase; TAF1: TATA-box binding protein associated factor 1; TAOK1: TAO kinase 1; TAOK2: TAO kinase 2; TGFBR2: transforming growth factor beta receptor 2; TNIK: TRAF2 and NCK interacting kinase; TRIO: trio Rho guanine nucleotide exchange factor; WNK1: WNK lysine deficient protein kinase 1; and EMT: epithelial-mesenchymal transition.

The majority of intracellular signaling pathways involving protein kinases with oncogenic potential start with receptor tyrosine kinases. Their activating phosphorylation initiates a cascade of reactions relayed predominantly by mitogen-activated protein kinase (MAPK) signaling of either ERK1/2, JNK, or p38 pathways, the phosphoinositide 3 kinase (PI3K)/Akt/mammalian (or mechanistic) target of rapamycin (mTOR) pathway PI3K/AKT/mTOR signaling, or the Janus kinase/signal transducer and activator of transcription (JAK/STAT) signaling. Their targets are as diverse as the ligands of upstream receptors, but ultimately control directly or indirectly via regulating the expression of genes involved in cell cycle progression, cell growth and differentiation, cellular metabolism, cytoskeleton organization, DNA repair and replication, and other cellular events, dysregulation of which renders a cell tumorigenic. Key pathways regulated by protein kinases, overexpression, translocation, point and fusion mutations, or simply dysregulation, which serve as a driver for the oncogenic transformation of the cell [9,17,22,23,24,27] are depicted in Figure 2.

A common feature of kinase proteins is the presence of a catalytic domain comprised of about 250 amino acids and preserved among the kinase types. Regulatory and other-than-catalytic domains are variable, allowing for a diversity of protein kinases [31]. Both the drug-targetable regions in that backbone and the generic structures of the inhibitors capable of binding to those target sites have been elucidated [21,28]. Most protein kinase inhibitors contain one or more heterocyclic moieties in their structure that can explain the difference in their binding to the target and thus the spectrum of activity among various compounds. The common heterocyclic moieties include quinazoline, quinoline, isoquinoline, pyridine, pyrimidine, pyrazole, benzimidazole, indazole, imidazole, indole, carbazole, or their fused structures [28]. This type of information provides a foundation for in silico analyses of natural products for kinase-specific inhibitory activities.

Therefore, the cellular kinome as a regulatory node of cellular physiology is one of the main foci of pharmacological drug discovery and respective therapeutic strategies aimed at cancer treatment, exemplifying an excellent potential for broader chemopreventive translation.

## 3. Protein Kinase Inhibitors at the Crossroads of Pharmacognosy and Chemotaxonomy

The American Society of Pharmacognosy defines *pharmacognosy* as “the study of the physical, chemical, biochemical, and biological properties of drugs, drug substances of natural origin as well as the search for new drugs from natural sources” [12]. While the term is not new, first being used between 1811 and 1815, an overlap of contemporary advances in de jure separate disciplines of pharmaceutical chemistry, pharmacobotany, pharmacology and toxicology of natural substances, phytochemistry, production of biogenic materials, the technology of natural drugs, and others, are encompassed de facto by pharmacognosy [32]. Out of 1881 newly approved drugs between 1981 and 2019, 18.4% were macromolecules of biological origin (large peptide or protein isolated or biotechnologically produced in cell lines or organisms), 3.8% originated from unaltered natural products, 18.9%—were from a natural product with semisynthetic modification, 11%—contained pharmacophores derived from natural products, and 0.8% were botanically defined mixtures of natural products according to the D.J. Newman and G.M. Cragg [33]. As a separate subcategory, the authors distinguished protein kinase inhibitors as natural product mimics able to competitively displace ATP. Altogether, the authors reported that 64.9% of drugs approved in the last 40 years contained natural products, whereas 36.3% out of 1394 discovered small molecule drugs were of natural origin. At the same time, 25% of the latter directly or indirectly originated in plants, in contrast to microorganisms providing 13% and animals 3% thereof [34]. The first written records of plants being utilized empirically for medical purposes was around 2600 BC in Mesopotamia, but rational clinical investigation of their properties in the context of drug discovery started at the beginning of the ninth century with the isolation of morphine from the opium of poppies (*Papaver somniferum*) in Germany [35]. Therefore, pharmacognosy remains at the base of the modern drug discovery process, and plants continue to be a popular source for the development of novel drugs.

### 3.1. Phytochemicals

Phytochemicals are the primary and secondary metabolites derived from biosynthetic processes underlying growth, development, and reproduction in every plant [36]. Each category of metabolite, i.e., primary and secondary, is likely to play complementary roles in efforts to provide protein kinase inhibitors to a select group of individuals using a culturally acceptable delivery vehicle. Primary metabolites include carbohydrates, amino acids, lipids, and nucleic acids. They exist in many complex conformations that affect both the ability to digest them to absorbable structures and also affect the ability of microorganisms to assimilate them as fuel sources [37]. Microbial access to these macromolecules as fuel sources is determined by their digested macronutrient’s primary, secondary, and tertiary structure. This is important because various microbial species are known to be sources of highly specific protein kinase inhibitors [38]. Consequently, if a natural product plant homogenate is used as a delivery vehicle, the macronutrients contained therein could indirectly exert effects via microorganisms that grow in the intestine in response to the plant homogenate.

Secondary metabolites generally exist in plants bound to other molecules, for example, carbohydrates, lipids, and proteins, affecting their bioavailability and the microbial species that occupy the intestinal tract. Plant secondary metabolites fall within a wide range of chemical classes and are estimated to include over 10,000 different chemicals [39]. Details of the interactions of microbes with both primary and secondary phytochemicals have recently been reviewed [40,41]. Many botanical families of plants are sources of protein kinase inhibitors that target various classes of protein kinase [38]. It is estimated that approximately only 15% of known existing plant species have been explored phytochemically, whereas reportedly, no more than 6% pharmacologically [35].

**Figure 3:**
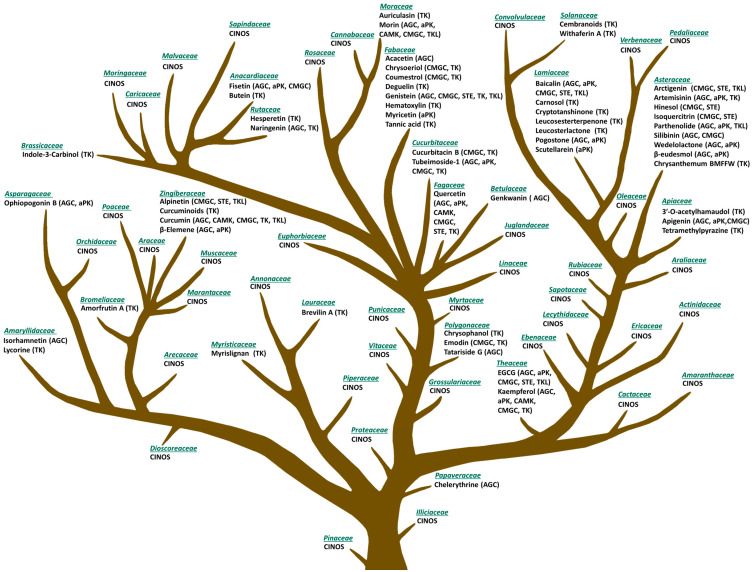
Evolutionary Tree of Plant-Derived Small Molecule Inhibitors. Adapted from [47,48] and supplemented with natural products with protein kinase-inhibiting properties using information from the literature [38,43,45,49,50,51,52,53,54,55,56,57,58,59,60]. Phytochemicals indicate only the botanical families where they were first discovered from. Other families still contain compounds identified but are not an original source (CINOS). More information is available in Supplementary Table S4.

Several exemplars are provided (Figure 3). Flavonoid apigenin found in vegetables like parsley, celery, and cilantro poses as an attractive natural product in both chemoprevention as well as combinatory therapy as it has been shown to reduce toxicity and sensitize the targets of currently applied chemotherapeutic drugs. It inhibits the activity of protein kinases within PI3K/AKT, JAK/STAT signaling cascades, CDK4/6, and CK2, as well as exhibits proapoptotic properties [38,42,43]. Clinical trials with legume-derived genistein showed a promising reduction in endometrial hyperplasia and its comorbidities by targeting VEGFR, EGFR, TGFBR, and apoptosis regulators [44]. Additionally, this isoflavone inhibits SRC, MAP2K4, and other protein kinases (Figure 3). The level of clinical studies also reached pyrrolophenanthridine alkaloid lycorine and sesquiterpene lactones with a peroxide bridge—artemisinins [45]. Artemisinin and its derivatives artemether, arteether, sodium artelinate, sodium artesunate, and many others, affect tumor immunosuppression, metastasis, angiogenesis, as well as metabolism, proliferation, apoptosis, oncosis, ferroptosis, and autophagy of cancer cells. Among other reported effects, they reduce expression of VEGF; ubiquitin-specific processing protease 33 (USP33); transforming growth factor β (TGF-β); and Ras-like B (RalB), Nanog, Oct3/4, ALDH1, CD44 and Sry-related high mobility group box (SOX2); as well as inhibit phosphorylation of EGFR, STAT3, PI3K, AKT, and mTOR [45,46].

### 3.2. Botanical Families

The conceptual organizational structure that we propose is based on a redeployment of the Evolutionary Tree of Plant-Based Foods that we originally published [47] and recently updated [48] (Figure 3). The tree was designed using principles of chemotaxonomy, the classification of plants based on similarities and differences in biochemical composition [36,38,43,45,49,50]. Accordingly, the rationale for developing a botanical evolutionary tree identifying protein kinase inhibitors is based on the premise that botanically distinct plants, as depicted in Figure 3, contain diverse phytochemicals and that these differences can be rationally exploited for medicinal purposes [48]. The probability of chemical similarities among plants derived from the same botanical family is greater than among plants classified in different botanical families [39].

The approach we propose for selecting plants for further drug discovery is chemosystematic and is complementary to ethnopharmacological (based on traditional medicine), ecological (taking into account interactions between plants and their environment), and computational (predicting bioactivity of plant metabolites in silico) approaches [35]. The botanical tree provides a chemotaxonomic resource for targeted drug development. An initial inspection of the tree provides many insights, three of which are (1) an evolutionary view of plant families that are rich sources of protein kinase inhibitors, (2) botanical families that have yet to be interrogated for small molecule inhibitors, and (3) a rational approach to choosing complementary botanical families for the prevention of a particular type of cancer-based on the driver kinases that are involved in the carcinogenic process, i.e., precision oncology. Thus, since many potentially significant bioactive phytochemicals remain to be identified, botanical groupings may provide direction for both protein kinase-specific inhibition as well as complementary inhibition of driver protein kinases in a particular type of cancer that belongs to different classes of protein kinases. To our knowledge, leveraging the principles of reverse pharmacognosy (from phytochemicals to plants producing them [61]) to systematically categorize plant material for the content of protein kinase inhibitors and the targets for which they have specificity is novel.

### 3.3. Pharmacological Considerations

Targeting plants as a source for novel pharmacological drug development does present many challenges. Accessibility and identification of plant biomaterial encompass difficulties of modern plant taxonomy (fluctuating systematics, nuances of genetic, chemical, morphological, anatomical characterizations, and disagreement thereof) and processing of its bioactives (harvesting and extraction technical challenges, stability and amount of phytochemicals). Other issues include growing and storage conditions, agricultural (impractical and loss during harvesting, lack of cultivation), ecological (unsustainable collection techniques), political (inter-country cooperation), and legal (failure to comply with the United Nation’s Convention on Biological Diversity) [35,62]. Natural products, in general, tend to possess low water solubility, higher molecular weight, and limited chemical stability [38,49]. Thus, pharmaceutical companies tend to show limited enthusiasm in targeting naturally produced chemicals for their commercial drug development. However, this creates a great platform for channeling phytochemicals toward chemoprevention through culturally accepted practices [35].

**Figure 4:**
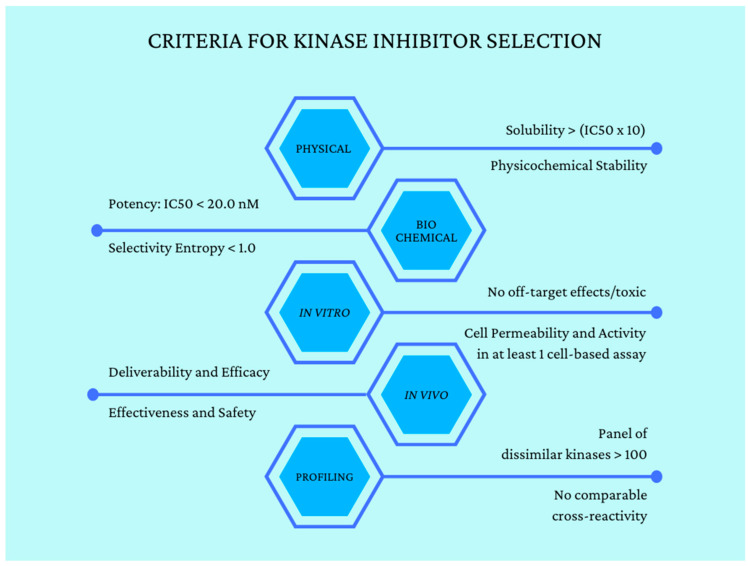
Standards for screening compound libraries for protein kinase inhibitor activity. Adapted from [64].

Drug development of protein kinase inhibitors and their clinical evaluation are extensive and provide a rich source of information by which to gauge the potential effectiveness of natural product-sourced protein kinase inhibitors. This includes data on plasma concentrations, whole-body partitioning, half-life, IC_50_, and target resident times that have been shown to render clinical benefit. There are also data from the drug literature on off-target effects, which could also be factored into potential effects of small molecule inhibitors from natural products [63]. Figure 4 summarizes standards that are used in screening compound libraries for protein kinase inhibitor activity [64]. These data are quite amendable to machine learning applications and, to our knowledge, such efforts have not received significant attention, at least in the public sector [65].

Phytochemical ATP-competitive inhibitors tend to have weaker affinity and selectivity for reversible compared with irreversible flavonoid analogues, e.g., genistein, hematoxylin, versus bacteria- or fungi-derived calphostin C, wortmannin, halenaquinol, and hypothemycin [38,49]. This poses a significant obstacle in considerations for pharmaceutical therapeutic selection of particular phytochemicals but is favorable for chemopreventative purposes as such weaker effects upon regular delivery may protect cells from acquiring oncogenic potential.

In terms of theoretical constructs, clonal expansion of transformed cells depends on the type of driver gene that was mutated, intra- and extracellular conditions, and respective cancer cell type. Therefore, a cancer cell can exhibit oncogene addiction, whereby the survival of a cancer cell depends strictly on the continuous oncogenic signaling orchestrated by the driver mutation. Alternatively, non-oncogene addiction leads a cancer cell to rely on the constitutive activity of non-mutated genes that support the transformed cell through the acquired cellular stresses. Consequently, in addition to the canonical objective to counteract or eliminate the effect of the driver mutation, cancer therapy can also aim to introduce non-compatible driver mutation in case of oncogene addiction to induce synthetic lethality of the cell, introduce additional cellular stress, or eliminate the activity of supporting genes, both of which would render the cell even more imbalanced and thus non-viable in terms of non-oncogene addiction [66,67]. Chemopreventative tailoring of protein kinase inhibitors from natural products can, therefore, prevent the clonal expansion of cancer cells, which despite net advantage, are more sensitive to cellular perturbations compared to normal cells.

### 3.4. Mode of Delivery

As mentioned above, the goal of the initiative advanced herein is not to discover new candidate natural products for drug development, although that would be of interest and benefit. The objective is rather to discern plant sources and plant combinations that can be given to individuals with specific types of cancer using etiological, environmental, lifestyle, and, to the extent feasible in the applicable healthcare system, diagnostic information to tailor a natural products cocktail to target relevant protein kinases to interference with disease progression. The term natural products “cocktail” is used to indicate that the delivery vehicle must be culturally appropriate for the target population. This, in turn, will inform critical issues relative to the bioaccessibility of the protein kinase inhibitors and whether effects on Phase I and Phase II metabolism need to be considered for establishing delivery dose and frequency.

## 4. Challenges and Opportunities

At least three factors identified in drug development in this field detract from the approach that has been presented. They are lower affinity of the natural products inhibitor for the target kinase(s) than achievable via synthesized drugs, the development of resistance to the effects of the inhibitor, and off-target effects of the inhibitor. Such concerns are legitimate, but beg the question: *“Is it better to do nothing or to work to maximize benefit despite the limitations noted?”* Relative to the stated limitations, it is noteworthy that many of the inhibitors currently in use in the clinic are promiscuous yet effective. Regarding resistance, it is recognized to occur in cancer because of the genomic instability of cells within a tumor. One strategy that has been explored in drug development to avoid resistance is the use of promiscuous protein kinase inhibitors—a fact that also relates to the third stated limitation, the occurrence of off-target activity (discussed in [28]).

By moving forward with the approach outlined herein, it is possible that mixtures of natural products could synergize and mitigate mechanisms of resistance by inadvertently targeting more than one relevant kinase. Another possibility is that multiple mechanisms could be targeted, as discussed below. Of particular interest, as illustrated by ongoing clinical trials in chronic lymphocytic leukemia, is that the protein kinase inhibitor, ibrutinib, is being combined with the BH3 mimetic inducer of apoptosis, venetoclax (ClinicalTrials.gov Identifier: NCT02910583). The rationale is that combining an inhibitor of cell proliferation with an inducer of apoptosis will exert synergistic cancer-specific cell kill. The results of Phase I–III clinical trials support this premise [68]. Conceptually, it is instructive to consider that an organism’s homeostasis is potent and dynamic, but this balance can be skewed towards new conditions that facilitate the onset of the multi-step process of carcinogenesis. D. Hanahan et al. emphasize enabling characteristics, such as genomic instability, non-mutational epigenetic reprogramming, inflammation, and polymorphic microbiota, as cellular and molecular mechanistic determinants of acquiring cancer hallmark capabilities initiating an oncogenic transformation of the cell and driving its further progression in the context of malignancy [22,23,24]. Consistent exposure of the organism to numerous carcinogenic risk factors promotes the development of such enabling characteristics. In the realm of this review, the intracellular kinome network orchestrates both oncogenic and tumor suppressor processes. If passive exposure to risk factors tunes the activity of these protein kinases into granting cancer hallmark capabilities, then active exposure to phytochemicals that inhibit their activity can balance the skewing homeostasis back. This embodies the essence of chemoprevention. For example, if tobacco smoking is associated with increased PI3K activity and the resulting development of lung cancer [69], then exposure of smokers to hibiscone C, quercetin, or myricetin has the potential to protect them from carcinogenesis. Similarly, the consumption of green tea and its component EGCG has been reported as a potent chemopreventive agent in cancer care [70]. Considering that cancer prevention spans exposure to a carcinogen (primary prevention), initiation, promotion, and progression of cancer (secondary prevention), and invasion and metastasis (tertiary prevention) [71], natural inhibitors of protein kinases can be chosen according to the type of cancer and/or the type of applied care and therapy with personalization and precision. Especially considering that many phytochemical compounds had chemosensitizing properties, increasing the effectiveness of chemotherapeutic drugs.

### 4.1. Beyond Protein Kinase Inhibition

The objective of small molecule inhibitor-mediated therapy is to reduce or eliminate the activity of proteins whose dysregulated function drives the carcinogenic process. While most targeted small molecules have been designed to inhibit their enzymatic activity via competition, covalent binding, or allosteric interference, the other type of target is their receptor, on which the small molecules exert either agonistic or antagonistic activity. However, the range of protein functions that are targeted and the processes they regulate are likely to expand as the field matures [28]. Two processes that are being targeted with small molecules that are of particular interest are apoptosis and immune checkpoint regulation.

#### 4.1.1. Targeting Apoptosis

Many types of cell death are operative in determining the balance between cell proliferation and cell death within the population of cells in a tumor [72]. Of these, evasion of apoptosis, a cancer hallmark, has been effectively targeted using small molecule BH3 mimetics that neutralize the antiapoptotic activity of Bcl-2 family members. This strategy is in clinical use and has been highly effective in arresting the progression of hematologic malignancies. Other positive and negative regulators of caspases, the cysteine proteases that serve as signaling mediators involved in orchestrating apoptotic execution pathways by proteolytically cleaving subsets of cellular proteins, are inhibitors of apoptosis (IAP) family proteins, cytochrome c, and second mitochondria-derived activator of caspases (SMAC), also known as Direct IAP-binding Protein with low pI (DIABLO) [72,73]. Balancing their regulation in transformed cells presents a physiological yet effective way to eliminate proliferative potential and reduce the progression of cancer. Natural products that modulate apoptosis have recently been reviewed [74].

#### 4.1.2. Targeting Immune Checkpoints

Tumors evolve to avoid immune attacks. The tumor microenvironment is immunosuppressive [75]. PD-1, PD-L1, and CTLA-4 are the three most popular immune targets [76]. PD-1 is a member of the CD28 family and is an inhibitory receptor expressed on activated T cells, B cells, macrophages, regulatory T cells (Tregs), and natural killer (NK) cells. It has two binding ligands, PD-L1 and PD-L2, expressed on normal cells. The combination of PD-1 with either of the ligands can inhibit T cell activity and induce T cell tolerance. Immunotherapy aims to inhibit the activity of these immune checkpoint blockers. The most significant benefit of immune checkpoint inhibitor therapy is using the immune function to destroy tumors. The field initially focused on developing antibodies to inactivate these molecular inhibitors of immunity, but the approach is expensive and associated with significant side effects. Consequently, the focus is shifting to small molecule inhibitors of these proteins that are orally administered and can penetrate cell membranes to act in cells [77]. CA170 was the first to obtain a new drug research application for small molecule immune checkpoint inhibitors [78]. CA-170 is the only small molecule modulator that can be taken orally for PD-1 and VISTA pathways and is an immune activation negative checkpoint modulator. It is under clinical investigation (ClinicalTrials.gov Identifier: NCT02812875). This is a rapidly emerging area because of the transformative nature of activation of the tumoricidal activity of the immune system at all stages of the carcinogenic process. The immunomodulatory potential of natural products used in herbal medicine has recently been reviewed [79].

## 5. Conclusions

As presented herein, chemoprevention mediated via selected protein kinase inhibitors of natural product origin and delivered using culturally appropriate “vehicles” in the context of precision oncology could provide a means to offer benefits to segments of the global population to which leading-edge therapies are unavailable. This small molecule-based approach has the potential to reach beyond protein kinase inhibitors to other mechanism-driven strategies including but not limited to apoptosis and immune checkpoint regulators. While the value of using highly specific drugs is clear, it is essential to leverage the science underlying current advances in cancer treatment to benefit individuals with limited opportunities to access the rapidly emerging advances in cancer prevention and control.

## Data Availability

Not applicable.

## Supplementary Materials

The following supporting information can be downloaded at: https://www.mdpi.com/article/10.3390/nu15051192/s1, Table S1: Kinase Drivers; Table S2: Cancer Associations; Table S3: Available Kinase Inhibitors; Table S4: Phytochemicals.
